# Burden of malaria infection among individuals of varied blood groups in Kenya

**DOI:** 10.1186/s12936-022-04251-1

**Published:** 2022-09-01

**Authors:** Redemptah Yeda, Charles Okudo, Eunice Owiti, Gladys Biwot, Cliff Momanyi, Winnie Korir, Thoya Mitsanze, Caroline Tegerei, Dennis Juma, Benjamin Opot, Edwin Mwakio, Gladys Chemwor, Raphael Okoth, Douglas O. Ochora, Agnes C. Cheruiyot, Amanda Roth, Hoseah M. Akala, Ben Andagalu

**Affiliations:** 1 Malaria Drug Resistance Laboratory, United States Army Medical Research Directorate-Africa (USAMRD-A), Kenya Medical Research Institute (KEMRI), Kisumu, Kenya; 2 Department of Plant Sciences, Microbiology & Biotechnology, College of Natural Sciences, Makerere University, P.O. Box 7062, Kampala, Uganda; 3 Walter Reed Army Institute of Research, Silver Spring, USA

**Keywords:** ABO, *Plasmodium falciparum*, Malaria, Blood group

## Abstract

**Background:**

The ABO blood groups consist of A, B, and H carbohydrate antigens, which regulate protein activities during malaria infection in humans. Understanding the interplay between the malaria parasite and blood group antigens is essential in understanding new interventions to reduce the global burden of malaria. This study assessed the burden of malaria infection among individuals with varying blood groups seeking treatment at selected hospitals in Kenya.

**Methods:**

A total of 366 samples from an ongoing malaria surveillance study were diagnosed for malaria by microscopy and further typed for blood group using ABO blood grouping. Age and sex were recorded in a data sheet, and analysed using R software version 4. Groups’ proportions (blood group, malaria infection, age and sex) were compared using Pearson’s Chi-square and Fischer exact tests. Wilcoxon and Kruskal-Wallis tests were performed and P-value < 0.05 was considered significant after Bonferroni correction for multiple comparisons. To understand the effect of each blood group on parasitaemia, multivariate logistic regression was used to model ABO blood group in relation to parasitaemia.

**Results:**

Of the 366 samples analysed, 312 were malaria positive, mean age was 9.83 years (< 5 years n = 152 (48.41%), 6 to 17 years n = 101 (32.16%) and > 18 years n = 61 (19.43%)). Malaria prevalence was higher among females than males, 54.46% and 45.54%, respectively. Kisumu enrolled the highest number 109 (35%)) of malaria cases, Kombewa 108 (35%), Malindi 32 (10%), Kisii 28 (9%), Marigat 23 (7%), and Kericho 12 (4%). Blood group O^+^ was the most prevalent among the enrolled individuals (46.50%), A^+^ (27.71%), B^+^ (21.02%) and AB^+^ (4.78%) respectively. Compared to blood group O+, blood group B^+^ individuals were (14%) were more likely to habour *Plasmodium falciparum* infection as opposed to A^+^ and AB^+^ individuals, that were 7% and 20%, respectively,. Those living in malaria-endemic zones presented with higher parasite densities compared to those living in malaria-epidemic (p = 0.0061). Individuals bearing B + blood group are more likely to habour high parasitaemia compared to O + blood group bearers (OR = 4.47, CI = 1.53–13.05, p = 0.006).

**Conclusion:**

Individuals of blood group B harbour high parasitaemia compared with the blood group O, Additionally, blood group A and B present with symptoms at lower parasitaemia than blood group O. Regardles of malaria transmission zones, individuals from endemic zones showed up with high parasitaemia and among them were more individuals of blood groups A and B than individuals of blood group O. Implying that these individuals were more at risk and require additional attention and effective case management.

**Garphical Abstract:**

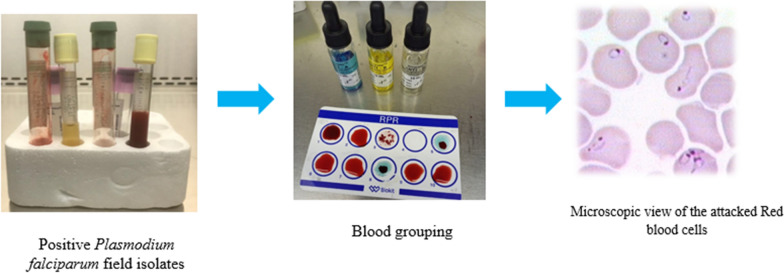

**Supplementary Information:**

The online version contains supplementary material available at 10.1186/s12936-022-04251-1.

## Background

The study of blood group antigens and malaria parasites is decades old, but new advances continue to be made that influence the understanding of interactions between malaria parasites and their human hosts. Malaria parasites spend a substantial part of their life cycle invading red blood cells (RBCs) and growing within them [1]. The invasion of RBCs is mediated by parasite adhesins and binding receptors on the red cell surface alongside a repertoire of signaling pathways within the erythrocyte, which alters red cell biophysical properties to and facilitate invasion [2]. Studies on RBC factors during the process of parasite invasion show that specific receptor-ligand interactions facilitate RBC binding involved in blood group antigens [3]. Parasite invasion rate into erythrocytes is dependent on both erythrocyte blood group antigen and haemoglobin gene namely; haemoglobin C (Hbc) and haemoglobin E (HbE) have been identified to confer varying susceptibility to *Plasmodium falciparum* infection [4].

Studies on the role of human genetic factors and diseases show an association between human susceptibility to malaria and the ABO blood group [5]. A systematic review and meta-analysis of 1923 articles obtained from the five databases by Degarege and coworkers showed an increased severity of *P. falciparum* infection among individuals with blood groups A, B, and AB in comparison with those of blood group O [6]. Specifically, blood groups A, B, and AB delay clearance of parasitized RBCs by promoting rosetting and cytoadherence, while blood group O increases clearance of RBC by reducing rosetting and cytoadherence [7]. However, there are divergent observations across regions. A similar study conducted in Mumbai, India found that people with blood group O are more prone to malaria infection in endemic areas [8]. Conversely, another study conducted among inhabitants of Odoakpu area of Onitsha in Anambra in Nigeria reported malaria to be most prevalent in individuals with blood group AB [9]. In Ethiopia, Tekese showed that patients with blood group O had a reduced chance of developing *P. falciparum* malaria as compared to patients with other blood groups [10]. Similar findings have been reported in India citing the vulnerability of non-blood group O and the protective advantage of blood group O [11].

The ABO blood groups have exerted evolutionary pressure [12, 13] in humans resulting in the natural selection of numerous polymorphisms in the genes encoding for erythrocyte surface proteins, haemoglobin, and immunity [14, 15]. The malaria-associated deaths in endemic areas have been shown to favor selection and retention of individuals bearing infection-resistant genetic variant erythrocytes affecting *Plasmodium* species invasion and replication within the RBC [16]. These divergent observations call for region-specific characterization of malaria alongside blood groups. This study reports on the burden of malaria infection among individuals with varying blood groups presenting at six hospitals located in 4 distinct malaria transmission zones across Kenya with uncomplicated malaria.

## Methods

### Subject enrolment and sample collection

Samples were collected between 2018 and 2020 from 6 hospitals located in 4 distinct malaria transmission zones across Kenya. The study enrolled individuals aged six months and above, presenting at outpatient departments with symptoms of malaria and/or testing positive for uncomplicated malaria by rapid diagnostic test (RDT; Parascreen® (Pan/Pf), Zephyr Biomedicals, Verna Goa, India) after providing written informed consent or assent. The demographic data and vital signs of each participant were entered in a case report form. 5 mL of venous blood was collected for RDT testing, smear preparation, blood group typing as well as the assessment of other study outcomes including sterile parasite culture. All malaria positive cases were treated with artemether-lumefantrine (AL)(Coartem®) according to the case management guidelines for uncomplicated malaria recommended by the Kenya Ministry of Health. Immediately after the blood draw, the attending clinician administered and directly observed taking the first dose of AL based on the patient’s weight. Each patient was given the remainder of the full dose of AL and advised to take the next dose after eight hours then follow up with the remaining doses at 12 hourly intervals till completion of the dose. Further, they were encouraged to return to the hospital should symptoms persist. Individuals with recurrent parasitaemia during the study period were treated but not re-enrolled.

### Malaria diagnosis

The number of parasites in each sample was estimated by examining Giemsa-stained thin and thick blood smears in 200 high-power fields. For quantification, parasite density per microlitre was estimated from the number of parasites counted per 2000 RBCs[18].

### ABO typing

ABO blood group typing was done as described by Olsson et al. [19]. One drop of whole blood from each participant was placed in three different spots on a grease-free clean glass slide. Three drops of blood group A, B, and Rhesus factor (D) anti-sera were applied onto each of the three different spots on the glass slide. The blood cells and the antigens were mixed with an applicator stick to homogeneity. The slide was then tilted to detect any agglutination and the results were recorded accordingly [20].

### Blood typing

There are four main blood groups defined by the ABO system. Blood group A has **A** antigens on the RBCs with anti **B** antibodies in the plasma, blood group **B** has **B** antigens on the RBCs and **A** antibodies in the plasma, blood group **O** has no antigens but has both anti **A** and anti **B** antibodies in the plasma and blood group **AB** has got both **A** and **B** antigens had no antibodies. If agglutination is observed when an individual’s blood is mixed with anti A reagent then the individual is said to have blood group “**A**”. If agglutination is observed when an individual’s blood is mixed with anti B reagent then the individual is said to have blood group “**B**”. If agglutination is observed when individuals blood is mixed with anti A and anti B reagent then the individual is said to have blood group “**AB**” and If no agglutination is observed when individuals blood is mixed with anti A and anti B reagent then the individual is said to have blood group “**O**”.

### Data management and analysis

Data showing blood group, parasite density, and demographics of each participant was entered in Microsoft Excel, checked for its correctness and exported for analysis using R software Version 4. Malaria parasite density was standardized by converting the values as percentages. Chi-square (χ2) was used to determine the association between blood groups and malaria infection. The difference between means was analysed using the Students t-test and one-way ANOVA test. P-values < 0.05 was considered significant.

### Ethics statement

This study was approved by the Kenya Medical Research Institute (KEMRI) and Walter Reed Army Institute of Research (WRAIR) institutional review boards protocol numbers KEMRI 3628 and WRAIR 2454, respectively.

## Results

### Demographic characteristics of the study participants

A total of 366 participants were enrolled in the study. After screening, 312 (84.25%) were found positive for *Plasmodium* sp, while 53 (25.75%) were negative for *Plasmodium* sp. Since the study aimed to assess the association between the burden of malaria infection and ABO blood groups, only the positives were used in analysis. The demographic characteristics of the 312 participants are summarized in Table 1. The participants were picked from different facilities allowing for comparisons between malaria-endemic, epidemic and seasonal areas. The various ABO blood groups were comparable across the below 5 years and above 5 years, and between the males and females.

**Table 1:** Demographic characteristics of the study participants

Characteristic		Total	ABO Blood Group				*P-value*
			A+	AB+	B+	O+	
Participants		312	87 (28%)	15 (5%)	66 (21%)	144(46%)	
Facility	Kericho level 5 Hospital	12	3 (25%)	1 (8%)	4 (33%)	4 (33%)	
	Kisumu level 5 Hospital	109	31 (28%)	4 (4%)	23 (22%)	51 (47%)	
	Kombewa level 4 Hospital	108	26 (24%)	8 (7%)	23 (22%)	51 (47%)	
	Kisii level 5 Hospital	28	4 (14%)	2 (7%)	12 (43%)	10 (36%)	
	Malindi level 4 Hospital	32	15 (47%)	0 (0%)	1 (3%)	16 (50%)	
	Marigat level 4 Hospital	23	8 (35%)	0 (()%)	3 (13%)	12 (52%)	**0.036** ^**b**^
Sex	Male	143	45	6	38	80	
	Female	169	42	9	28	64	0.606^a^
Age Groups	< 5 years	151	47	6	28	80	
	> 5 years	161	40	9	38	64	0.247^a^

Bold text indicates a statistically significant difference with a *P-value* < 0.05. Groups’ proportions were compared using ^a^Pearson’s Chi-square and ^b^Fischer exact tests

### **The burden of*Plasmodium*infection**

**Fig. 1:**
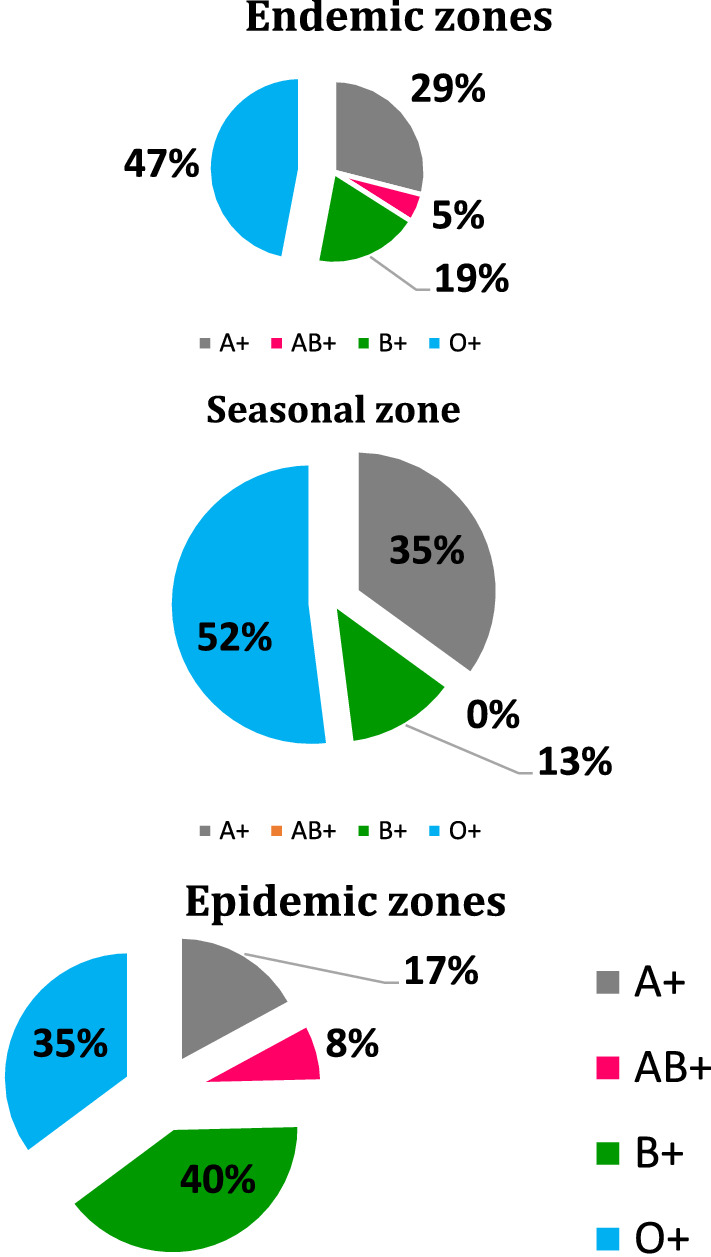
Blood group representation in the three malaria zones

**Fig. 2:**
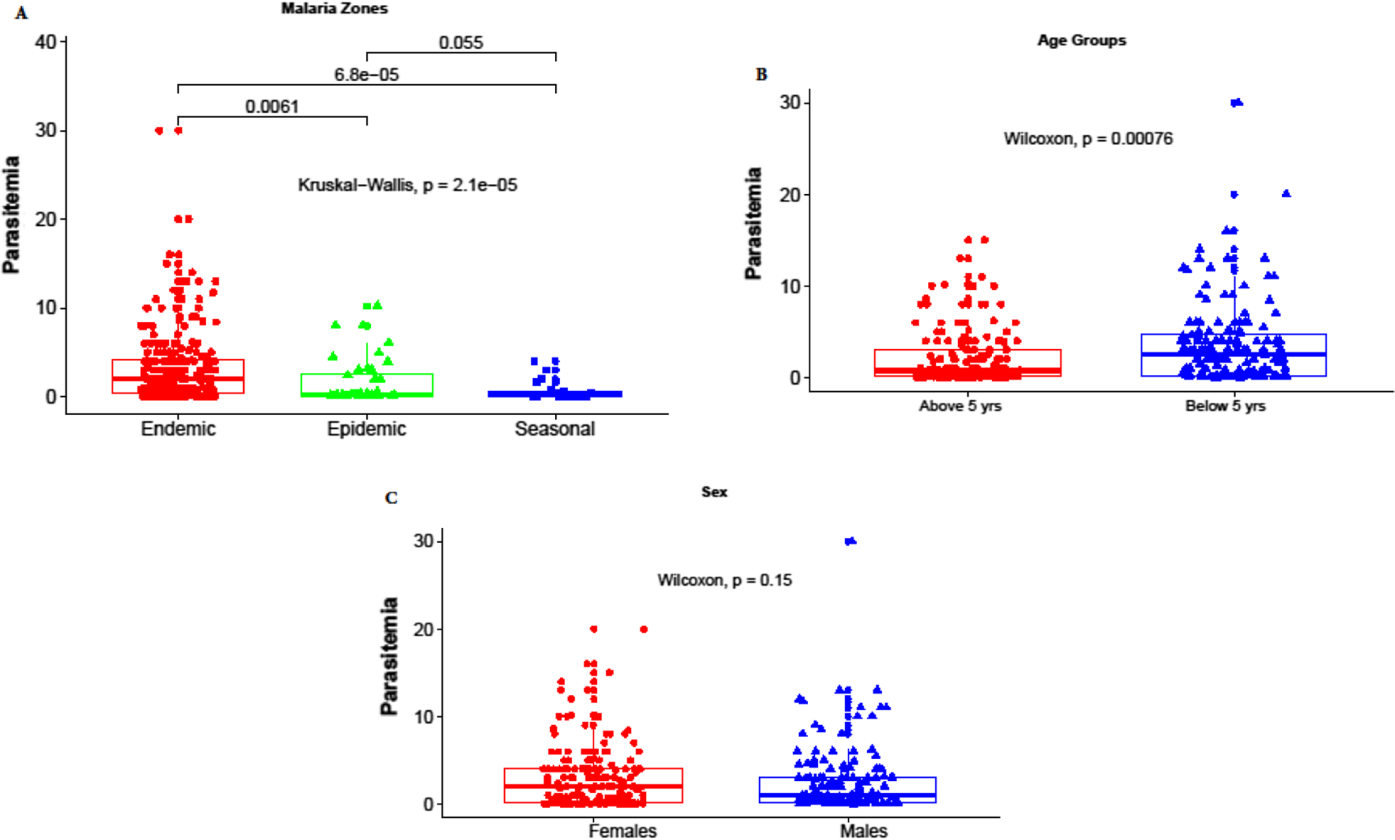
Red and blue colour represent parasite densities in different malaria zones, age groups and gender

Parasitaemia level is a known measure of the burden of *Plasmodium* infection in the population [18]. A comparison of blood groups in relation to malaria zones is depicted in Fig. 1. Those living in malaria-endemic zones present with higher parasite densities compared to those living in seasonal malaria zones (p = 0.0061) and malaria seasonal zones (*p = 6.8e-05*). The results are consistent with other studies showing high parasite densities in malaria-endemic regions [21]. A comparison in parasitaemia was done between the younger and the older participants and showed patients above 5 years old had the lowest parasite density (p = 0.00076). The levels of parasite densities between the males and the females were however comparable (p = 0.15) (Fig. 2).

### ABO blood group and parasitaemia

Factors associated with parasitaemia levels are important in creating a model for ABO Blood groups’ relationship with parasitaemia as shown in Table 2. Parasitaemia levels were arbitrariliy characterized as low when the number of parasites per 2000 RBC in the three fields counted are below 1%, moderate when when the number of parasites per 2000 RBC in the three fields counted are between 2%and 9%, and high when when the number of parasites per 2000 RBC in the three fields counted are equal to above 10%. Malaria zones were significantly associated with parasitaemia (p = 0.002) with endemic regions leading with parasitaemia levels. Further, age groups were also associated with Parasitaemia levels (p = 0.0006) and the above 5 years leading with low parasite densities. Finally, ABO blood group system was also associated with parasitaemia levels (p = 0.011), so we sought to understand the effect of each blood group on parasitaemia.

**Table 2:** Factors influencing the parasitaemia levels

	Total	Parasitaemia l evels			*P value*	OR^c^ (95% CI)	*P Value*
		High	Moderate	Low			
N	312	21 (6%)	108 (35%)	183 (59%)			
Malaria Zones	Endemic	20(8%)	96 (9%)	133 (53%)		7.64 (1.58–36.95)	**0.012** ^**c**^
	Epidemic	1 (2%)	10 (25%)	29 (73%)		1.32 (0.20–8.72)	0.774^c^
	Seasonal	0 (0%)	2 (9%)	21 (91%)	**0.002** ^**b**^	**1 (Ref)**	
Age Groups	< 5 Years	14 (9%)	72(48%)	65(43%)		2.752 (1.21–6.28)	**0.016** ^**c**^
	> 5 Years	7(4%)	43(27%)	111(69%)	**0.0006** ^**a**^	**1 (Ref)**	
ABO Blood Group	A+	6 (7%)	29 (33%)	52 (60%)		1.25 (0.27–5.79)	0.717^c^
	AB+	3 (20%)	2 (13%)	10 (67%)		1.19 (0.46–3.12)	0.289^c^
	B+	9 (14%)	24 (36%)	33 (50%)		4.47 (1.53–13.05)	**0.006** ^**c**^
	O+	3 (2%)	53 (37%)	88 (61%)	**0.011** ^**a**^	**1 (Ref)**	

*CI* confidence interval,* OR* odds ratio,* Ref* reference,* N* Number of Participants. Bold text indicates a statistically significant difference with a *P-value* < 0.05. Groups’ proportions were compared using ^a^Pearson’s Chi-square, ^b^Fisher exact tests and ^c^Multivariate logistic regression. ^c^OR obtained in multivariate logistic regression adjusting for all factors in the table

A model was created to show association between parasitaemia and ABO blood group (Table 2). There are chances that individuals bearing some blood groups can be harbouring high parasitaemia. From this model, individuals living in malaria-endemic zones are more likely to harbour high parasitaemia as compared to those living in malaria seasonal zones (OR = 7.64, CI = 1.58–36.95, p = 0.012). Participants below 5 years are more likely to harbour high parasite densities (OR = 2.752, CI = 1.21–6.28, p = 0.016). Lastly, individuals bearing B + blood group are more likely to harbour high parasitaemia compared to the O + blood group bearers (OR = 4.47, CI = 1.53–13.05, p = 0.006) (Additional file 1).

## Discussion

Studies on host blood groups and malaria are inconclusive given the variability in malaria geographic distribution of infections and the need to characterize each of the ecotypes. This study showed that individuals from endemic zones reported with symptoms at higher parasitaemia than individuals from seasonal zones regardless of blood groups. Additionally, blood group A and B presented at lower parasitemia than blood group O in either of the transmission regions. Previous studies have reported that semi-immunity to malaria infection develops based on the cumulative exposure to the parasite [23]. These findings suggests that distribution of ABO blood group in humans may have had a direct influence by selecting genetic pressure from *P. falciparum* infection. Thus again, implying these individuals require additional attention hence effective case management (Additional file 2).

This study has showed that individuals of blood group B are 4 times more likely to harbour high parasitaemia followed by A then AB individuls. Data reveals that this individuals will show up in hospitals with very low parasitaemia, suggesting that individuals of such blood groups are the ones at high risk of fatal outcome of malaria than the individuals of blood group O. Similar studies have been reported in Nigeria revealing evidence that malaria infection was most prevalent in individuals with blood group AB [9]. This data suggests additional attention and effective case management should be directed towards individuals of these blood group that show up at the health facilies with lower parasitaemia more frequently.

This observation gives credence to findings from a study demonstrated in Douala Cameroon, that showed blood group A and B individuals were more predisposed to malaria infections than blood group O+ [21]. Findings from this study concur with other studies from Ethiopia, Srilanka, and Gabon in which individuals of blood group A and B were more prone to malaria infection compared to blood group O [22, 23]. These evidence suggests that malaria burden could be shaping population of individuals or vise verser.

This study however had limitations. This study lacked information on other RBC polymorphisms that have been associated with clinical outcomes of malaria, e.g. glucose-6-phosphate dehydrogenase, complement receptor-1 and haemoglobinopathies. Secondly, the study inclusion criteria ensured that only individuals who had positive tests for uncomplicated malaria were included, therefore uninfected individuals could not be characterized as a control group.

## Conclusion

The findings highlighted that individuals of blood group A and B present with symptoms at lower parasitaemia than individuals of blood group O. This study showed that individuals of blood group A and B still present with high parasitaemia than individuals of blood group O. This implies that individuals of these blood groups were more at risk of fatal outcome of malaria than those of blood group O hence they required extra effective case management.

Center Lines represent medians, with lower and upper boundaries of the boxes representing first and third quartiles respectively. Wilcoxon and Kruskal-Wallis tests were performed and *P-value* < 0.05 was considered significant after Bonferroni correction for multiple comparisons.

## Supplementary Information

**Additional file 1.** Distribution of malaria infection among various malaria zones in relation to blood group,sex and gender.

**Additional file 2.** Analysis to compare parasitemia to various blood groups, sex and gender across different malaria zones.

## Data Availability

Not applicable.
