# Supplementary material for: Burden of malaria infection among individuals of varied blood groups in Kenya
Source: Malar J. 2022 Sep 1;21:251. doi: 10.1186/s12936-022-04251-1 (PMC9438094; doi:10.1186/s12936-022-04251-1)
Supplement: Supplementary file 1 — Additional file 1. Distribution of malaria infection among various malaria zones in relation to blood group,sex and gender. [file 12936_2022_4251_MOESM1_ESM.pdf]

```
Redempta=read.csv(file.choose(),header=T)

#Check data structure
str(Redempta)

#Creating Groups based on the parasitenia levels
Parasetemia_levels=cut(Redempta$Parasitemia,breaks = c(0,2,9,300),labels =
c("low","moderate","high"))
levels(Parasetemia_levels)

#Create groups based on the aes
Age_groups=cut(Redempta$Age,breaks = c(0,5,100),labels = c("Below 5 yrs","Above 5 yrs"))
levels(Age_groups)

#Ceate a final data set
Redempta=cbind(Redempta ,Parasetemia_levels)
Redempta=cbind(Redempta ,Age_groups)

#Check data structure
str(Redempta)

#Export data to csv
write.csv(Redempta,"C:/Users/Edwin Agwati/Documents/R/Redempta/Redempta_F.csv",row.names =
F)

#Change variables
Redempta$Sex=as.factor(Redempta$Sex)
Redempta$Age_groups=as.factor(Redempta$Age_groups)
Redempta$Parasetemia_levels=as.factor(Redempta$Parasetemia_levels)
```

```
Redempta$Group=as.factor(Redempta$Group)

Redempta$Facility=as.factor(Redempta$Facility)

Redempta$Malaria.Zones=as.factor(Redempta$Malaria.Zones)

Redempta$Age=as.numeric(Redempta$Age)


#Check data structure

str(Redempta)


#Costructing a contingency table for age roups and parasitemia levels

table.1=table(Redempta$Parasetemia_levels,Redempta$Age_groups)

table.1


#chisquare test of independance:  Checks if the probability distribution of one variable is affected by the
presence of another.

chisq.test(table.1)


#Costructing a contingency table for parasitemia levels and blood groups

table.2=table(Redempta$Parasetemia_levels,Redempta$Group)

table.2


#chisquare test of independance:  Checks if the probability distribution of one variable is affected by the
presence of another.

chisq.test(table.2)


#Load libraries

library(ggplot2)

library(ggpubr)


#Check data structure
```

```
str(Redempta)


#ggplots for the comparison between Age groups

ggboxplot(Redempta, x = "Age_groups", y = "Parasitemia",

          color = "Age_groups", palette = c("red", "blue"),

          add = c("jitter", "boxplot"), shape = "Age_groups",

          title = "Below and Above 5",

          ylab = "Parasitemia", xlab = "Age Groups",

          font.y = c(14, "bold"),

          font.legend = c(14, "bold", "black"))+

  stat_compare_means(label.x = 1.3, label.y = 16, size = 4) + theme(plot.title = element_text(size=16, face="bold", hjust = 0.5), axis.title.y = element_text( size=10, face="bold"), axis.title.x =element_text( size=10, face="bold"))



#ggplots for the comparison between Males and Females

ggboxplot(Redempta, x = "Sex", y = "Parasitemia",

          color = "Sex", palette = c("red", "blue"),

          add = c("jitter", "boxplot"), shape = "Sex",

          title = "Males Vs Females",

          ylab = "Parasitemia", xlab = "Sex",

          font.y = c(14, "bold"),

          font.legend = c(14, "bold", "black"))+

  stat_compare_means(label.x = 1.3, label.y = 16, size = 4) + theme(plot.title = element_text(size=16, face="bold", hjust = 0.5), axis.title.y = element_text( size=10, face="bold"), axis.title.x =element_text( size=10, face="bold"))


#Prepare a list of comparisons

my_comparison=list(c("Endemic","Epidemic"), c("Endemic","Seasonal"), c("Epidemic","Seasonal"))
```

```
#ggplots for the comparison across Malaria zones

ggboxplot(Redempta, x = "Malaria.Zones", y = "Parasitemia",
        color = "Malaria.Zones", palette = c("red", "blue", "green"),
        add = c("jitter", "boxplot"), shape = "Malaria.Zones",
        title = "Comparison across Malaria Zones",
        ylab = "Parasitemia", xlab = "Malaria Zones",
        font.y = c(14, "bold"),
        font.legend = c(14, "bold", "black"))+
  stat_compare_means(comparisons = my_comparison) + stat_compare_means(label.x = 1.3, label.y = 16, size = 4) + theme(plot.title = element_text(size=16, face="bold", hjust = 0.5), axis.title.y = element_text( size=10, face="bold"), axis.title.x =element_text( size=10, face="bold"))


#Logistic regression

logistic=lm(Redempta$Parasitemia ~ relevel(Group, ref = "O+") + relevel(Age_groups, ref = "Below 5 yrs") + relevel(Malaria.Zones, ref = "Endemic"), data = Redempta)

summary(logistic)


exp(coef(logistic))

exp(cbind(OR = coef(logistic), confint(logistic, level = 0.95)))
```
