# Supplementary material for: Burden of malaria infection among individuals of varied blood groups in Kenya
Source: Malar J. 2022 Sep 1;21:251. doi: 10.1186/s12936-022-04251-1 (PMC9438094; doi:10.1186/s12936-022-04251-1)
Supplement: Supplementary file 2 — Additional file 2. Analysis to compare parasitemia to various blood groups, sex and gender across different malaria zones. [file 12936_2022_4251_MOESM2_ESM.pdf]

Facility	Parasitemi	Group	Age	Sex	Malaria Zones
KCH	8	A+	6	F	Epidemic
KCH	0.295	B+	19	F	Epidemic
KCH	6	B+	3	F	Epidemic
KCH	0.16	AB+	27	F	Epidemic
KCH	2	O+	1	M	Epidemic
KCH	0.1	B+	14	F	Epidemic
KCH	3	A+	1	M	Epidemic
KCH	0.5	O+	2	M	Epidemic
KCH	10.2	O+	22	F	Epidemic
KCH	8	O+	19	F	Epidemic
KCH	3.9	B+	1	F	Epidemic
KCH	4.45	A+	6	F	Epidemic
KDH	0.3	A+	12	F	Endemic
KDH	0.3	O+	14	M	Endemic
KDH	4	A+	1	F	Endemic
KDH	3	O+	2	F	Endemic
KDH	0.00241	O+	24	F	Endemic
KDH	8.6	O+	21	F	Endemic
KDH	4	O+	24	F	Endemic
KDH	9	B+	4	F	Endemic
KDH	6	O+	6	F	Endemic
KDH	0.00241	B+	1	M	Endemic
KDH	4	O+	9	M	Endemic
KDH	0.1	A+	34	M	Endemic
KDH	2.7	O+	2	F	Endemic
KDH	2	B+	5	F	Endemic
KDH	6	A+	3	F	Endemic
KDH	12	B+	3	F	Endemic
KDH	5	AB+	5	F	Endemic
KDH	3	B+	2	F	Endemic
KDH	0.1	O+	5	F	Endemic
KDH	0.2	O+	3	F	Endemic
KDH	12	B+	1	M	Endemic
KDH	0.01	O+	2	F	Endemic
KDH	0.6	O+	4	M	Endemic
KDH	3	A+	3	F	Endemic
KDH	3	O+	10	F	Endemic
KDH	3.6	B+	3	F	Endemic
KDH	1.6	A+	4	F	Endemic
KDH	0.02	A+	3	F	Endemic
KDH	2	A+	1	M	Endemic
KDH	2	O+	10	M	Endemic
KDH	0.01	O+	38	M	Endemic
KDH	3	O+	9	M	Endemic
KDH	2.12	B+	18	M	Endemic
KDH	6.2	O+	6	M	Endemic

KDH	0.008	O+	15	M	Endemic
KDH	1	O+	1	M	Endemic
KDH	1	A+	8	M	Endemic
KDH	4	O+	5	F	Endemic
KDH	0.1	B+	2	F	Endemic
KDH	4.5	O+	1	M	Endemic
KDH	0.03	O+	3	M	Endemic
KDH	1.93	O+	2	F	Endemic
KDH	6	O+	5	F	Endemic
KDH	0.077	B+	3	M	Endemic
KDH	6	O+	5	M	Endemic
KDH	3.1	A+	4	M	Endemic
KDH	1.33	O+	5	F	Endemic
KDH	1	O+	11	M	Endemic
KDH	0.0002	A+	3	F	Endemic
KDH	13	B+	15	M	Endemic
KDH	0.1	AB+	13	M	Endemic
KDH	2	B+	2	M	Endemic
KDH	2	O+	2	M	Endemic
KDH	5	A+	9	F	Endemic
KDH	1	O+	22	M	Endemic
KDH	0.8	B+	1	F	Endemic
KDH	0.01	B+	7	F	Endemic
KDH	0.04	B+	2	M	Endemic
KDH	3.7	O+	3	F	Endemic
KDH	0.4	O+	23	F	Endemic
KDH	3	O+	2	F	Endemic
KDH	0.6	O+	21	F	Endemic
KDH	20	AB+	4	F	Endemic
KDH	0.7	B+	23	F	Endemic
KDH	0.2	O+	6	M	Endemic
KDH	1	O+	23	F	Endemic
KDH	15	B+	6	F	Endemic
KDH	0.06	A+	41	F	Endemic
KDH	0.05	A+	2	F	Endemic
KDH	0.5	O+	10	M	Endemic
KDH	0.2	O+	3	M	Endemic
KDH	4	B+	11	M	Endemic
KDH	30	B+	3	M	Endemic
KDH	3	O+	3	M	Endemic
KDH	3	B+	8	F	Endemic
KDH	0.3	A+	33	F	Endemic
KDH	2.4	A+	24	F	Endemic
KDH	8.4	A+	3	F	Endemic
KDH	0.2	O+	24	F	Endemic
KDH	0.001	A+	3	M	Endemic
KDH	0.2	A+	12	M	Endemic

KDH	2.58	A+	22	M	Endemic
KDH	13	A+	3	M	Endemic
KDH	2	O+	16	F	Endemic
KDH	8	B+	26	M	Endemic
KDH	4	O+	3	F	Endemic
KDH	1	A+	21	F	Endemic
KDH	0.7	O+	3	F	Endemic
KDH	4	A+	21	F	Endemic
KDH	4	A+	17	F	Endemic
KDH	4.1	A+	4	M	Endemic
KDH	0.6	A+	9	M	Endemic
KDH	2.5	O+	20	M	Endemic
KDH	0.014	A+	30	F	Endemic
KDH	4	A+	9	F	Endemic
KDH	2	AB+	5	M	Endemic
KDH	0.16	A+	6	M	Endemic
KDH	1	O+	73	F	Endemic
KDH	5	O+	1	F	Endemic
KDH	3	O+	1	M	Endemic
KDH	0.2	B+	2	M	Endemic
KDH	2.6	A+	4	F	Endemic
KDH	0.08	B+	3	M	Endemic
KDH	5.2	A+	6	F	Endemic
KDH	0.015	O+	40	F	Endemic
KDH	0.85	O+	21	M	Endemic
KDH	0.4	O+	22	M	Endemic
KDH	1	O+	40	F	Endemic
KDH	1	O+	34	M	Endemic
KOM	5	B+	4	M	Endemic
KOM	13	A+	5	F	Endemic
KOM	10	AB+	2	M	Endemic
KOM	14	B+	4	F	Endemic
KOM	0.003	O+	4	F	Endemic
KOM	5	O+	2	F	Endemic
KOM	2	B+	5	F	Endemic
KOM	0.006	A+	4	F	Endemic
KOM	3	O+	5	F	Endemic
KOM	10	A+	5	F	Endemic
KOM	6	B+	3	F	Endemic
KOM	5	O+	3	M	Endemic
KOM	3	O+	3	F	Endemic
KOM	0.02	O+	3	F	Endemic
KOM	4	O+	5	F	Endemic
KOM	11	O+	4	M	Endemic
KOM	4	O+	2	F	Endemic
KOM	0.7	A+	2	F	Endemic
KOM	6	O+	3	M	Endemic

KOM	11	A+	3	M	Endemic
KOM	7	B+	4	F	Endemic
KOM	0.42	O+	6	F	Endemic
KOM	2	O+	14	F	Endemic
KOM	2	A+	24	F	Endemic
KOM	4	O+	4	F	Endemic
KOM	1	O+	13	M	Endemic
KOM	2	B+	4	M	Endemic
KOM	4	A+	1	F	Endemic
KOM	4	B+	5	F	Endemic
KOM	2.1	O+	3	M	Endemic
KOM	4.1	B+	1	F	Endemic
KOM	1.66	O+	10	F	Endemic
KOM	2.47	B+	4	F	Endemic
KOM	3.59	O+	9	F	Endemic
KOM	0.046	A+	7	M	Endemic
KOM	0.0069	A+	4	M	Endemic
KOM	16	A+	4	F	Endemic
KOM	0.7	AB+	21	M	Endemic
KOM	0.13	AB+	3	F	Endemic
KOM	8.5	O+	2	M	Endemic
KOM	0.012	B+	14	F	Endemic
KOM	9	O+	5	F	Endemic
KOM	13	B+	5	F	Endemic
KOM	9	O+	1	M	Endemic
KOM	0.03	A+	21	F	Endemic
KOM	0.7	AB+	3	F	Endemic
KOM	3	AB+	2	M	Endemic
KOM	6	O+	6	F	Endemic
KOM	0.046	O+	4	F	Endemic
KOM	3	O+	1	M	Endemic
KOM	0.9	A+	5	M	Endemic
KOM	0.07	O+	1	M	Endemic
KOM	3	O+	1	F	Endemic
KOM	4	B+	5	M	Endemic
KOM	10	B+	7	F	Endemic
KOM	0.02	A+	5	F	Endemic
KOM	0.031	O+	6	M	Endemic
KOM	0.01	B+	6	F	Endemic
KOM	10	B+	6	F	Endemic
KOM	0.03	O+	7	M	Endemic
KOM	2.9	O+	2	M	Endemic
KOM	0.17	O+	6	M	Endemic
KOM	7	O+	3	F	Endemic
KOM	3	B+	2	M	Endemic
KOM	3	A+	7	F	Endemic
KOM	3	O+	5	F	Endemic

KOM	2	O+	5	F	Endemic
KOM	2	O+	6	F	Endemic
KOM	4	B+	5	F	Endemic
KOM	3	A+	6	M	Endemic
KOM	6	O+	13	F	Endemic
KOM	0.9	O+	9	F	Endemic
KOM	0.003	B+	4	F	Endemic
KOM	0.8	O+	3	M	Endemic
KOM	6	O+	6	M	Endemic
KOM	2	O+	10	M	Endemic
KOM	6	O+	3	M	Endemic
KOM	1	B+	9	F	Endemic
KOM	0.009	B+	10	F	Endemic
KOM	4	O+	29	F	Endemic
KOM	10	A+	9	M	Endemic
KOM	0.046	O+	2	M	Endemic
KOM	5	B+	12	F	Endemic
KOM	0.006	A+	14	M	Endemic
KOM	0.64	O+	7	M	Endemic
KOM	0.046	O+	16	F	Endemic
KOM	0.24	AB+	2	M	Endemic
KOM	0.01	A+	3	M	Endemic
KOM	1.5	O+	5	M	Endemic
KOM	2.8	A+	4	M	Endemic
KOM	4.6	O+	3	M	Endemic
KOM	1	O+	20	F	Endemic
KOM	0.8	A+	29	F	Endemic
KOM	3.9	O+	11	M	Endemic
KOM	4.9	O+	4	M	Endemic
KOM	4.5	B+	2	M	Endemic
KOM	8	O+	9	F	Endemic
KOM	0.007	AB+	10	M	Endemic
KOM	4	A+	6	F	Endemic
KOM	2	B+	10	F	Endemic
KOM	1	A+	11	M	Endemic
KOM	11.7	O+	5	M	Endemic
KOM	0.009	A+	9	M	Endemic
KOM	2.6	A+	5	M	Endemic
KOM	6	A+	1	F	Endemic
KOM	4	O+	8	F	Endemic
KSI	0.08	A+	15	M	Epidemic
KSI	0.13	O+	28	F	Epidemic
KSI	0.13	B+	1	M	Epidemic
KSI	0.23	O+	4	F	Epidemic
KSI	0.07	O+	21	F	Epidemic
KSI	0.1	A+	22	F	Epidemic
KSI	0.2	B+	11	M	Epidemic

KSI	0.18	O+	3	M	Epidemic
KSI	0.086	AB+	18	M	Epidemic
KSI	0.1	O+	10	F	Epidemic
KSI	3	B+	2	M	Epidemic
KSI	0.15	B+	14	M	Epidemic
KSI	0.11	O+	5	F	Epidemic
KSI	0.3	B+	2	M	Epidemic
KSI	0.1976	AB+	2	F	Epidemic
KSI	0.1	O+	45	F	Epidemic
KSI	0.2	O+	18	M	Epidemic
KSI	0.08	O+	6	M	Epidemic
KSI	0.2	O+	3	F	Epidemic
KSI	0.06	A+	21	F	Epidemic
KSI	2	B+	6	F	Epidemic
KSI	0.2	B+	35	F	Epidemic
KSI	4.9	B+	1	M	Epidemic
KSI	2.4	B+	17	M	Epidemic
KSI	3.1	B+	7	M	Epidemic
KSI	0.2	A+	2	M	Epidemic
KSI	0.24	B+	2	F	Epidemic
KSI	0.23	B+	4	F	Epidemic
MDH	5	O+	4	F	Endemic
MDH	0.1	A+	5	M	Endemic
MDH	0.97	O+	1	F	Endemic
MDH	1.8	O+	30	F	Endemic
MDH	0.11	A+	13	M	Endemic
MDH	0.3	O+	12	M	Endemic
MDH	0.2	A+	3	M	Endemic
MDH	0.69	O+	1	F	Endemic
MDH	0.29	A+	22	F	Endemic
MDH	0.19	O+	3	M	Endemic
MDH	5.48	A+	5	M	Endemic
MDH	0.233	O+	10	M	Endemic
MDH	2	O+	51	M	Endemic
MDH	8	O+	10	F	Endemic
MDH	1	A+	4	F	Endemic
MDH	5	A+	12	F	Endemic
MDH	2	A+	3	F	Endemic
MDH	0.039	A+	18	M	Endemic
MDH	0.5	O+	13	F	Endemic
MDH	1.3	O+	15	M	Endemic
MDH	4.4	O+	12	M	Endemic
MDH	1.3	O+	42	F	Endemic
MDH	0.8	O+	27	F	Endemic
MDH	0.77	B+	23	F	Endemic
MDH	0.7	A+	3	M	Endemic
MDH	6	A+	8	F	Endemic

MDH	8	A+	6	M	Endemic
MDH	0.6	A+	9	M	Endemic
MDH	0.02	A+	20	M	Endemic
MDH	0.2	O+	52	F	Endemic
MDH	0.16	O+	29	F	Endemic
MDH	0.5	A+	3	M	Endemic
MGT	0.004	A+	2	M	Seasonal
MGT	0.38	O+	15	F	Seasonal
MGT	1.94	A+	12	M	Seasonal
MGT	4	O+	7	M	Seasonal
MGT	3	A+	16	M	Seasonal
MGT	0.08	A+	10	F	Seasonal
MGT	0.02	O+	9	F	Seasonal
MGT	0.1	A+	8	F	Seasonal
MGT	0.3	O+	5	F	Seasonal
MGT	0.18	B+	21	M	Seasonal
MGT	0.16	O+	10	M	Seasonal
MGT	0.08	O+	6	M	Seasonal
MGT	0.2	O+	9	F	Seasonal
MGT	0.7	O+	7	M	Seasonal
MGT	0.08	B+	21	M	Seasonal
MGT	0.39	A+	18	F	Seasonal
MGT	0.12	O+	9	M	Seasonal
MGT	0.014	A+	13	M	Seasonal
MGT	0.07	O+	7	F	Seasonal
MGT	0.06	B+	19	M	Seasonal
MGT	1.6	A+	3	M	Seasonal
MGT	0.06	O+	20	F	Seasonal
MGT	0.58	O+	21	F	Seasonal
KOM	1	A+	3	M	Endemic
KOM	11	AB+	6	M	Endemic
